# The GPR17 agonist galinex restores oligodendrocyte maturation under inflammatory conditions

**DOI:** 10.3389/fphar.2026.1838997

**Published:** 2026-06-05

**Authors:** Juliana Helena Castro e Silva, Davide Marangon, Marta Boccazzi, Stefano Raffaele, Nicolò Cignitti, Martina Bavetta, Ivan Arisi, Marta Fumagalli, Maria P. Abbracchio, Davide Lecca

**Affiliations:** 1 Department of Pharmaceutical Sciences, University of Milan, Milan, Italy; 2 Department of Pharmacological and Biomolecular Sciences, University of Milan, Milan, Italy; 3 European Brain Research Institute “Rita Levi-Montalcini”, Rome, Italy; 4 Institute of Translational Pharmacology - National Research Council (IFT-CNR), Rome, Italy

**Keywords:** GPR17, neuroinflammation, oligodendrocyte, oligodendrocyte precursor cell (OPC), pro-inflammatory cytokines

## Abstract

**Introduction:**

Chronic neuroinflammation disrupts oligodendrocyte differentiation and limits effective remyelination across multiple neurological disorders. Among the molecular regulators integrating inflammatory cues with oligodendrocyte maturation, G protein-coupled receptor 17 (GPR17) has emerged as a critical checkpoint. Physiologically, GPR17 expression is low in early oligodendrocyte precursor cells (OPCs), peaks in immature oligodendrocytes, and is subsequently downregulated to allow terminal maturation. Under neuroinflammatory conditions, GPR17 expression persists, suggesting a possible role in impaired oligodendrocyte maturation and defective myelination. Here, we tested whether receptor modulation by the selective GPR17 agonist Galinex (GAL) can support oligodendrocyte maturation under inflammatory conditions.

**Methods:**

Differentiating oligodendroglial cultures were exposed to a pro-inflammatory cytokine cocktail composed of TNFα, IL-1β, and IFNγ. We first identified a subtoxic inflammatory condition, defined as cytokine exposure that did not cause overt loss of cell viability, and assessed oligodendrocyte maturation, myelin-associated marker expression, GPR17 expression, and transcriptional remodelling. Publicly available transcriptomic signatures from neuroinflammatory mouse models and human Alzheimer’s disease and multiple sclerosis brains were used for cross-comparison. The effect of GAL was then evaluated by molecular, morphological, and functional readouts, including a synthetic nanofiber myelination assay.

**Results:**

Subtoxic cytokine exposure consistently impaired oligodendrocyte morphological maturation, reduced the expression of myelin-associated markers, and was accompanied by increased GPR17 expression. Transcriptomic analysis revealed coordinated remodelling of pathways related to protein synthesis and proteostasis, mitochondrial metabolism, lipid homeostasis, and inflammatory/immunogenic-like responses, together with senescence- and DNA damage-associated features. Cross-comparison with disease-associated transcriptomic signatures showed significant overlap with neuroinflammatory modules, supporting the relevance of the inflammatory pathways engaged in our model. GAL treatment partially restored terminal maturation-associated features and oligodendrocyte morphology. Moreover, in the nanofiber assay, GAL significantly increased the length of MBP-positive segments compared with CTK-treated cells, suggesting improved wrapping/myelination-like capacity after inflammatory challenge.

**Discussion:**

Together, this study establishes a controlled in vitro model linking inflammatory cytokine exposure, disease-associated transcriptional alterations, and impaired oligodendrocyte differentiation. Our findings indicate that pharmacological modulation of GPR17 can promote oligodendrocyte maturation and wrapping features under non-permissive inflammatory conditions. This strategy should be considered as an oligodendroglial-directed approach that may complement anti-inflammatory or immunomodulatory interventions.

## 1 Introduction

A growing body of evidence indicates that myelin and white-matter integrity are compromised across a range of neuroinflammatory and neurodegenerative disorders. In demyelinating diseases such as multiple sclerosis (MS), myelin loss is a defining pathological feature, while in conditions including Alzheimer’s disease (AD), Parkinson’s disease (PD), and amyotrophic lateral sclerosis (ALS), accumulating data support the contribution of oligodendroglial dysfunction and myelin abnormalities in disease progression (Klotz et al., 2023; Dean et al., 2016; Bryois et al., 2020). Damage to and loss of mature oligodendrocytes (OLs), the myelinating cells of the CNS, not only drives myelin disruption but can also amplify neuronal vulnerability by weakening axonal support and metabolic coupling (Molina-Gonzalez et al., 2022). Thus, therapeutic strategies aimed at supporting oligodendrocyte maturation may not only enhance endogenous remyelination but also mitigate secondary neuronal damage (Klotz et al., 2023).

OLs provide essential physical, metabolic, and trophic support to neurons, and successful remyelination relies on both mature OLs that survive demyelination and newly generated OLs arising from OPC differentiation (Kuhn et al., 2019; Baydyuk et al., 2020; Bacmeister et al., 2020). However, pathology-driven chronic inflammation creates a non-permissive environment that hinders the remyelination process (Macchi et al., 2020; Rodriguez et al., 2014; Neely et al., 2022; Franklin et al., 2021). Importantly, remyelination failure does not simply reflect a lack of progenitors, as OPCs can proliferate and accumulate around lesions but fail to complete differentiation. Although surviving mature OLs contribute to repair, OPC-derived newly generated OLs remain a major source of regenerated myelin and are therefore a critical target for pro-remyelinating strategies (Duncan et al., 2018; Yeung et al., 2019). This limited differentiation indicates an active blockade of their differentiation program, pointing to inflammatory and sensory cues as key regulators of oligodendroglial timing and fate decisions (Franklin et al., 2024). In neuroinflammatory conditions, impaired OL differentiation occurs within a sustained pro-inflammatory milieu generated by both resident and infiltrating immune cells. Activated microglia and astrocytes, together with infiltrating macrophages and T lymphocytes, release high levels of cytokines such as interleukin-1β (IL-1β), tumor necrosis factor-α (TNF-α), and interferon-γ (IFN-γ), which exert profound effects on oligodendroglial lineage cells (Harrington et al., 2020). IL-1β and TNF-α are predominantly produced by activated microglia and macrophages and act as central amplifiers of inflammatory signalling within the CNS, whereas IFN-γ is mainly derived from infiltrating T cells and prominently associated with inflammatory demyelinating diseases, including MS, but also reported in other inflammatory neurodegenerative contexts (Haroon et al., 2024). Exposure to these mediators has been shown to disrupt the OL differentiation program, suppressing myelin gene expression and terminal maturation. Together, these observations suggest that inflammatory cytokines do not simply damage oligodendroglia but interfere with the timing of molecular mechanisms that normally control the transition from OPCs to myelinating OLs, motivating the search for regulatory nodes that couple inflammatory signalling to differentiation dynamics.

A compelling candidate node is the G protein-coupled receptor 17 (GPR17), a receptor transiently expressed in OPCs that functions as a key checkpoint of the differentiation trajectory (Lewash et al., 2025). Under physiological conditions, GPR17 expression is tightly regulated, being transiently upregulated at early stages and subsequently downregulated to allow terminal maturation (Lecca et al., 2020). However, under diverse chronic neuroinflammatory conditions characterized by defects in myelination, including rodent demyelinating models (He et al., 2021; Coppolino et al., 2018; Bonfanti et al., 2020; Boda et al., 2011), and human neurological diseases (Angelini et al., 2021; Raffaele et al., 2025; Satoh et al., 2017), GPR17 temporal regulation is disrupted, with pathological persistence of receptor upregulation in oligodendroglial cells accumulating in and around demyelinating lesions and failing to acquire a fully myelinating phenotype. In this context, persistent GPR17 expression may contribute to, or reflect, the altered differentiation state of oligodendroglial cells exposed to inflammatory cues. Sustained engagement of GPR17 by agonists has been shown to trigger receptor desensitization and internalization (Fratangeli et al., 2013; Daniele et al., 2014; Fumagalli et al., 2015; Daniele et al., 2011), which, in turn, acts as an intracellular signal to initiate events involved in OL terminal maturation. On this basis, pharmacological modulation of GPR17 through ligands promoting its intracellular downregulation may represent an oligodendroglial-directed approach to facilitate oligodendrocyte differentiation despite persistent inflammatory signalling. Thus, targeting GPR17 is not intended to replace anti-inflammatory strategies, but to act on a lineage-specific differentiation checkpoint that may remain therapeutically relevant when inflammatory activity is only partially controlled.

In this study, we established an *in vitro* model of inflammation-induced OL differentiation impairment using a low, subtoxic concentration of a pro-inflammatory cytokine cocktail (CTK) relevant to multiple neuroinflammatory and neurodegenerative contexts. We performed global transcriptomic profiling of primary rat OPCs exposed to CTK to identify and confirm the molecular programs and pathways engaged during early inflammatory stress. We then investigated whether pharmacological modulation of GPR17 signalling with the selective agonist galinex (GAL) can counteract inflammation-associated differentiation failure and restore OL maturation. Together, these findings provide a mechanistic framework to dissect GPR17-dependent regulation of oligodendrocyte differentiation under inflammatory conditions and support its potential as a target to promote remyelination.

## 2 Materials and methods

### 2.1 Animals

All procedures were approved by the Italian Ministry of Health (License code 5247B.N.L2Y). Animals were kept according to the local animal use committee and in accordance with the Guide to the Care and Use of Laboratory Animals of the National Institutes of Health and the European Community Council Directives. All experiments from the present study used both males and females wild-type Sprague-Dawley pups at the post-natal age 0–2 days (P0-P2).

### 2.2 Primary glial culture and oligodendrocyte isolation

For our investigation, we worked with primary oligodendrocytes obtained from a mixed glial rat primary culture as described previously (Chen et al., 2007), and modified by us (Fumagalli et al., 2011). Briefly, cortices from P0-P2 pups were dissected and placed in Hanks’ Balanced Salt Solution (HBSS) (ThermoFisher Scientific, 24,020–091). Tissue was washed and incubated in a Trypsin EDTA DNAse I (Sigma-Aldrich, D5025-150KU) solution to allow chemical dissociation, followed by mechanical dissociation and centrifugation. Cells were plated onto poly-_D_-lysine coated flasks (Euroclone, ET7076) in Dulbecco-modified Eagle Medium High Glucose (DMEM HG, Euroclone, ECB7501L) containing 1% penicillin-streptomycin (ThermoFisher Scientific, 15,140–122), 1% Glutamax supplement (ThermoFisher Scientific, 35050061), and 10% FBS. The medium of the flasks was changed every day *in vitro* (DIV) until they reached confluence (typically 8 DIV).

Upon confluence, flasks were shaken using an orbital shaker to remove microglia followed by an additional 3-h shaking to obtain OPCs. Cells were seeded in a density of 13,000–15,000 cells/cm^2^ onto poly-_D,L_-ornithine coated plates (Sigma Aldrich) and kept for 3DIV in proliferating conditions in oligodendrocyte medium composed of Neurobasal medium containing 1% penicillin-streptomycin, 1% Glutamax, 2% B27 supplement (ThermoFisher Scientific, 17504044), human platelet-derived growth factor β (Sigma-Aldrich, P3201), human basic fibroblast growth factor (R&D systems Minnesota, 233-FB), and N-acetylcysteine (Sigma-Aldrich, A8199). The purity of OPC-enriched cultures obtained with this standardized protocol has been previously characterized by our group, showing very limited contamination by non-oligodendroglial cells, mainly astrocytes ranging from 0% to 4%, with negligible microglial contamination (Lecca et al., 2016). To further verify culture purity in the present study, one OPC preparation used for the current experiments was immunostained for GFAP and Iba1. Quantification revealed 0.22% GFAP-positive cells and no detectable Iba1-positive cells among DAPI-positive cells (data not shown).

### 2.3 Treatments

Each cytokine–TNF-α, IL-1β and IFN-γ (Immunotools) – was resuspended in sterile PBS with 0.1% BSA at 100 μg/mL. The cytokine cocktail consisted of TNFα, IL-1β, and IFNγ. Three CTK conditions were tested and are referred to as CTK-2, CTK-6, and CTK-20. In CTK-2, each cytokine was used at 2 ng/mL; in CTK-6, each cytokine was used at 6 ng/mL; and in CTK-20, each cytokine was used at 20 ng/mL. Galinex (GAL, Ambinter, Orlénas, France, AMB9128576) was dissolved using dimethyl sulfoxide (DMSO, Sigma-Aldrich, Darmstadt, Germany, 4722301) to obtain 10 mM stock solutions. Both cytokines and GAL stock solutions were filtered with 0.22 µm filters in sterile conditions and kept in −20 °C as single-use aliquots.

All treatments were performed when cells were switched to differentiating condition (day in differentiation (DID) 0), with medium lacking growth factors and containing 5 nM of T3 (except for myelination assay onto nanofibers, where 15 nM T3 was added). Cells initially received ¾ of the volume of medium containing equal concentrations of each pro-inflamatory cytokine and, after 6 h, cells received the remaining ¼ of media containing media with four time-concentrated GAL or equivalent DMSO to reach the final desired concentration (10 nM). In myelination assay experiments GAL treatment was performed every other day and cells were fixed at DID7.

### 2.4 Immunocytochemistry

For immunocytochemistry staining, cells were plated onto poly-ornithine coated glass coverslips or onto synthetic nanofibers (NanoAligned®, Nanofiber Solutions 2,402). After each experiment endpoint, cells were fixed using 4% paraformaldehyde and sucrose (Sigma Aldrich 84,097) in PBS for 20 min. The coverslips or plate bottom were then washed thrice with PBS for staining. Immunostaining was performed as previously described (Marangon et al., 2022). Primary antibodies were incubated overnight as follows: anti-BCAS1/NABC1 (1:100, Rabbit, Fischer Scientific BS-1426R) and anti-myelin basic protein (MBP) (1:200, Rat, Sigma-Aldrich, MAB386). Primary antibody incubation was followed by three washes and secondary antibody incubation for 1 h at room temperature (Invitrogen AlexaFluor 1:800, goat) specific to the primary host species. Cell nuclei were stained using Höeschst 33342 (Invitrogen H21491, 1:10,000) and coverslips were mounted on glass slides using Dako Mounting Media (Agilent S3023).

### 2.5 Fluorescent imaging and analysis

For cell count, at least three coverslips were analysed from four independent experiments. After immunostaining, 30 images of random fields were acquired per coverslip using a fluorescence microscope (Zeiss Axiovert 200M). Cell count was performed considering the entire fields using the Cell count plugin from ImageJ Fiji software (Schindelin et al., 2012) (https://imagej.net/software/fiji).

Analysis of OL cytoskeleton complexity was performed using Neuroanatomy plugin as previously described (Facchinetti et al., 2022). For this, Z-stacks were acquired at a confocal microscope (Zeiss LSM900) and the MBP fluorescent channel of each image was converted into binary 8-bit images. Fifteen cells were analysed per coverslip, with a total of three coverslips per condition, each one from an independent experiment. Starting radius was set at 10 µm and step size (radii interval of consecutive circles) at 5 µm. Results are expressed in the maximum number of intersections per cell, number of intersections per concentric radius per cell, and cell diameter (µm).

For the myelination assay, wells were mounted on glass slides after the experiment endpoint, and images were acquired using confocal microscopy. Then, the myelinated segments (areas of straight alignment and colocalization between FITC-positive nanofibers and MBP-positive cell branches) from at least 8 cells per condition was measured using the freehand tool from at least five different spots at the orthogonal views of measured using the freehand measuring tool of ImageJ Fiji. Results are expressed in average length of myelinated segment per cell from two independent experiments.

### 2.6 qRT-PCR

After each experimental endpoints, cells were lysed using Trizol™ reagent (ThermoFisher Scientific, 15596026). RNA was extracted using the Direct-zol RNA Microprep Kit (ZymoResearch, R2062), then quantified and treated with DNase (Promega, M198A). Retrotranscription to cDNA was performed using SensiFAST™ cDNA Synthesis Kit (Meridian Bioscience, BIO-65054). qRT-PCR was performed using SYBR green technology (SensiFAST™ SYBR® No-ROX Kit (Meridian Bioscience), using previously validated rat primers listed in Supplementary Table S1. All procedures were performed according to manufacturer’s instructions in a CFX Connect Real-Time PCR Detection System (Bio-Rad). The analyses were performed using Rpl13a as housekeeping gene and the 2^-ddCT^ method for relative mRNA expression. Results were normalized to the average expression of each experiment control.

### 2.7 Next-generation RNA-sequencing and data analyses

RNA-sequencing (RNA-seq) was performed 24 h after adding GAL. For that, RNA of three replicates per group was isolated as described in section 2.6 and library preparation and sequencing were performed by Xenovea Kft (Szeged, Hungary). Quality control of raw sequencing reads was performed using FastQC. Genome indexation and alignment from the *Rattus norvegicus* reference was performed using STAR R package. A comprehensive filtering strategy was applied to improve data quality: unannotated features were removed, and low-abundance genes were filtered out using the “Average” method with an abundance threshold of 5. Additionally, to exclude invariable features, a variance filter was applied to remove the lowest 15% of features based on interquartile range. Following filtration, data normalization was performed using the Log2-counts per million (logCPM) transformation to adjust for sequencing depth and stabilize variance prior to downstream analysis. Differential expression analysis was performed with the DESeq2 package and differentially expressed genes (DEGs) were filtered based on |log_2_FC| ≥ 0.59 and adjusted p-value ≤0.05.

### 2.8 Functional enrichment analysis and visualization

Functional enrichment analysis was performed independently for the upregulated and downregulated gene lists using the g:Profiler web server (version e113_e.g.,59_p19, accessed on 21 January 2026). To ensure statistical accuracy, the analysis was conducted using a custom reference background consisting of all genes detected in the RNA-seq dataset. The query focused on Gene Ontology (GO) terms for Biological Process (BP) and Molecular Function (MF), as well as KEGG pathways. The significance threshold was set at an adjusted p-value <0.05 (g:SCS). Enrichment results were visualized as dot plots, generated in RStudio (version 2025.05.01) using the ‘ggplot2′ package. Additionally, to visualize the overlap and relationships between enriched terms, a Circos plot was generated using the Metascape platform.

### 2.9 Protein-protein interaction (PPI) network construction and module analysis

Protein-protein interaction (PPI) networks were constructed separately for upregulated and downregulated DEG (CTK vs. CTL) using the STRING database (version 12.0), applying a minimum confidence interaction score of 0.700. The resulting networks were visualized and analysed using Cytoscape (version 3.10.4). To identify functional protein complexes and dense modules, clustering analysis was performed using the Markov Clustering (MCL) algorithm via the *clusterMaker2* Cytoscape application. The inflation parameter was optimized for each dataset to improve cluster granularity: an inflation value of 3.0 was applied to the upregulated network, while a value of 2.0 was used for the downregulated network, using the STRING interaction score as edge weight. The top 10 largest subnetworks were extracted and subjected to functional enrichment analysis (Gene Ontology and KEGG pathways) to determine their biological significance, considering terms with an FDR <0.05 as significant.

### 2.10 Statistics

All statistics and plots were performed using GraphPad Prism 9.0.0 Software for Windows (GraphPad Software, Boston, Massachusetts USA, www.graphpad.com, Accessed on 21 December 2024), considering the average of three wells and at least three independent experiments. The averages of each experiment are shown as dots in the bar graphs, unless stated. Specific hypothesis testing tests are declared in figure legends. To all analysis, we admitted *p* < 0.05 as statistically significant.

## 3 Results

### 3.1 Establishing a model of inflammation-induced oligodendrocyte differentiation impairment

**FIGURE 1:**
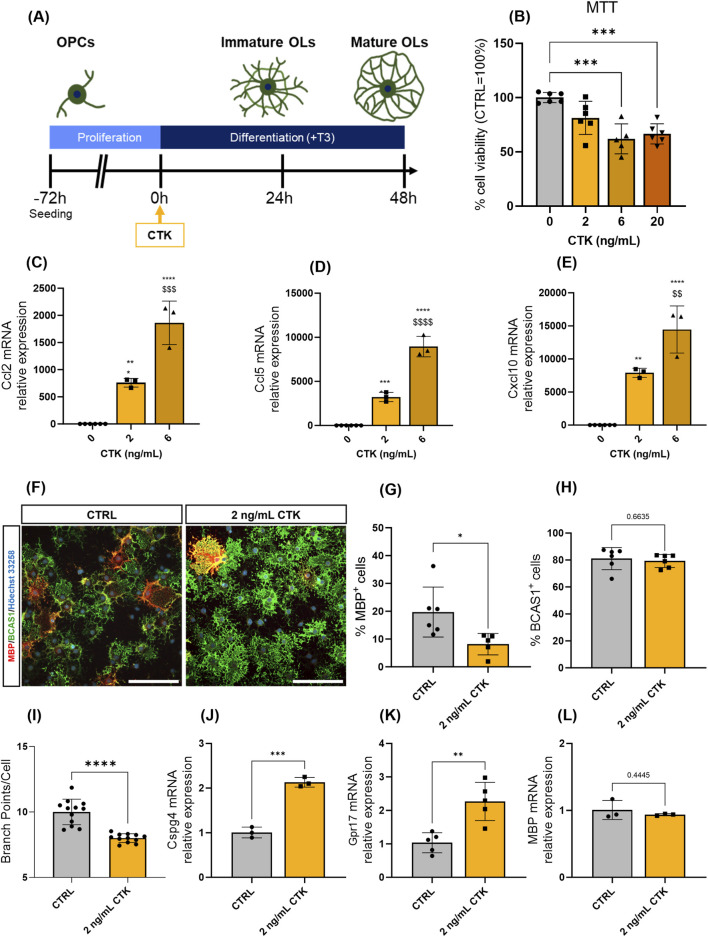
CTK-2 identifies a subtoxic cytokine condition that triggers inflammatory activation and impairs oligodendrocyte maturation. **(A)** Experimental design for characterizing a model of inflammatory oligodendrocyte differentiation impairment. Cells were kept for 3 days in proliferating conditions, then treated with different concentrations of TNF-α, IL-1β, and IFN-γ in pro-differentiating conditions. **(B)** Average MTT absorbance relative to untreated control after 72 h of treatment **(C–E)** qRT-PCR after 24 hours-treatment showing relative mRNA expression (2^-ddCt^) of the pro-inflammatory chemokines **(C)** CCL-2 **(D)** CCL-5 and **(E)** CXCL-10. Dots represent the average of each experiment normalized to the average of the control (CTRL = 1). **(F)** Representative images (scale bar: 50 µm) of the percentage of **(G)** MBP- and **(H)** BCAS1-positive cells. **(I)** Analysis of branch points number normalized on total cell count performed at 48 h post-treatment. **(J–L)** qRT-PCR showing relative gene expression of **(J)** Gpr17 and **(K)** Cspg4 and **(L)** Mbp gene expression after 48 h of treatment with 2 ng/mL CTK. All graphs show mean ± SD **(B)** One-way ANOVA with Bonferroni *post hoc*. **(C–E)** One-way ANOVA with Tukey *post hoc*. **(G–L)** Two-tailed unpaired t-test. In all graphs ‘*’ indicates p value classification when compared to control group and ‘$’ indicates p value classification when compared to CTK-2 group. *p < 0.05; **p < 0.01; ***p < 0.001.

To develop a model of inflammation-induced oligodendrocyte differentiation impairment, we exposed primary rat OPCs to a pro-inflammatory cytokine cocktail (CTK) containing TNF-α, IL-1β and IFN-γ. Three CTK conditions were tested and are hereafter referred to as CTK-2, CTK-6, and CTK-20, indicating that each cytokine in the mixture was used at 2, 6, or 20 ng/mL, respectively (Figure 1A). To select a concentration that induced inflammation without being cytotoxic, we performed a MTT viability assay at the end of 72 h of treatment. We observed that CTK-20 significantly reduced cell viability by 33.38% compared with the untreated control, and that CTK-6 had a similar effect (Figure 1B). On the contrary, CTK-2 exposure did not induce any significant reduction in cell viability. To assess whether CTK-2 could trigger an inflammatory response, we performed qRT-PCR for selected inflammatory markers 24 h after treatment. Both CTK-2 and CTK-6 induced a marked increase in the mRNA expression of the pro-inflammatory chemokines CCL-2, CCL-5, and CXCL-10 (Figures 1C–E), confirming the ability of CTK-2 to promote an inflammatory response in OLs. Based on these findings, we selected the non-toxic CTK-2 cocktail for subsequent experiments, since it was sufficient to elicit a significant degree of inflammation without compromising cell viability.

Next, we investigated whether CTK-2 exposure was sufficient to induce differentiation impairment. Immunofluorescence analysis 48 h after treatment showed that CTK-2 reduced the percentage of mature MBP-positive cells by approximately 40% (Figures 1F,G). Interestingly, the reduction in MBP-positive cells was not accompanied by a decrease in breast carcinoma amplified sequence 1 (BCAS1)-positive cells (Figures 1F,H), a marker of earlier stages of myelinating OLs (Fard et al., 2017), indicating a block at later differentiation stages. Consistently, analysis of the number of branch points per cell performed on the whole cell population highlighted a significant reduction of branch complexity (Figure 1I). Additionally, qRT-PCR revealed persistence of upregulation of Gpr17 as well as of Cspg4, which, in turn, encodes for the early OPC marker NG2 (Figures 1J,K), confirming a delay in OL maturation. However, there were no significant changes in the gene expression of the gene encoding for MBP at this time point (Figure 1L).

### 3.2 CTK-2 rewires OL gene networks toward immune activation and metabolic shutdown

**FIGURE 2:**
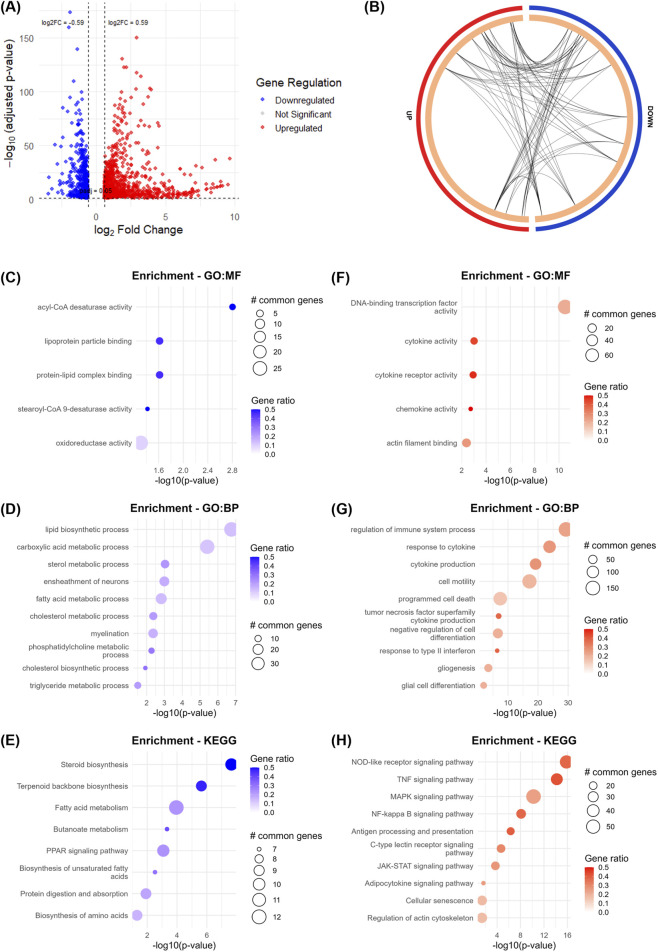
Pro-inflammatory cytokines drive immune activation and metabolic suppression in oligodendrocytes. **(A)** Volcano plot of differentially expressed genes in CTK-2 vs. CTL groups shown as log_2_ fold change versus −log_10_ (adjusted P value). Upregulated genes are shown in red and downregulated genes in blue. Dashed lines indicate the applied thresholds (|log_2_FC| ≥ 0.59 and adjusted P value ≤0.05). **(B)** Circular plot generated using the top 500 significantly upregulated (UP) and downregulated (DOWN) gene. Links indicate shared enriched terms, revealing functional convergence or separation. **(C–H)** Functional enrichment analyses performed separately on downregulated **(C–E)** and upregulated **(F–H)** gene sets. Dot plots report enriched Gene Ontology Molecular Function (GO:MF), Biological Process (GO:BP), and KEGG pathways. Dot size represents the number of genes associated with each term, color indicates gene ratio, and the x-axis shows enrichment significance expressed as −log_10_(P value).

To gain additional insights into the molecular mechanisms at the basis of CTK-2 exposure in OLs, we performed RNA sequencing 24 h after treatment. We obtained 1786 differentially expressed genes (DEGs) (Supplementary Table S2), from which 1,198 were upregulated and 588 downregulated (Figure 2A). A circular enrichment map generated from the top 500 up- and downregulated genes highlighted a marked functional segregation between the two transcriptional programs, with limited sharing of enriched terms (Figure 2B). Functional enrichment analysis of downregulated genes revealed a repression of molecules involved in lipid metabolism and myelination-associated processes after CTK-2 treatment (Figures 2C–E). Specifically, enriched terms included acyl-CoA desaturase activity and lipid/lipoprotein binding functions (Figure 2C). Consistently, biological process terms were mostly related to lipid biosynthetic and metabolic pathways (e.g., sterol/cholesterol metabolism, fatty acid and triglyceride metabolism, phosphatidylcholine metabolism), as well as oligodendrocyte-relevant categories such as myelination and ensheathment of neurons (Figure 2D). KEGG analysis further reinforced this signature, with strong enrichment for steroid biosynthesis, terpenoid backbone biosynthesis, fatty acid metabolism, biosynthesis of unsaturated fatty acids, and PPAR signalling (Figure 2E). In line with these pathways, the downregulated set included multiple genes encoding rate-limiting enzymes involved in fatty acid and cholesterol synthesis (e.g., Fasn, Scd2, Acsl1/3/4, Fads1/2/3, Hmgcr, Dhcr7, Dhcr24, Nsdhl, Sqle). These findings provide molecular confirmation that, in our CTK-based *in vitro* model, inflammatory exposure hinders oligodendrocyte maturation at least in part by suppressing lipid anabolic programs required for membrane expansion and myelin production.

In parallel, enrichment analysis of upregulated genes showed a robust induction of immune- and cytokine-associated pathways (Figures 2F–H). Upregulated molecular functions included cytokine activity, chemokine activity, cytokine receptor activity, and DNA-binding transcription factor activity (Figure 2F). Enriched biological processes included terms such as regulation of immune system process, response to cytokine, cytokine production (including TNF superfamily cytokine production), cell motility, programmed cell death, and response to type I interferon; notably, gliogenesis and glial cell differentiation terms also emerged within the upregulated set, consistent with a broad remodelling of glial-state programs under inflammatory stress (Figure 2G). KEGG pathways enriched among upregulated genes included NOD-like receptor signalling, TNF signalling, NF-κB signalling, MAPK signalling, C-type lectin receptor signalling, JAK–STAT signalling, and antigen processing and presentation (Figure 2H). Interestingly, “Cellular senescence” was also among the enriched KEGG terms (Figure 2H). Consistent with this signal, senescence/DNA-damage–associated regulators within the DEG list (e.g., Tp53, Rras, Serpina1) were strongly upregulated, together with cell-cycle regulators such as Ccnd1 and Cdk6, whose induction in post-mitotic cells is commonly interpreted as markers of stress-associated cell-cycle dysregulation and DNA-damage responses.

**FIGURE 3:**
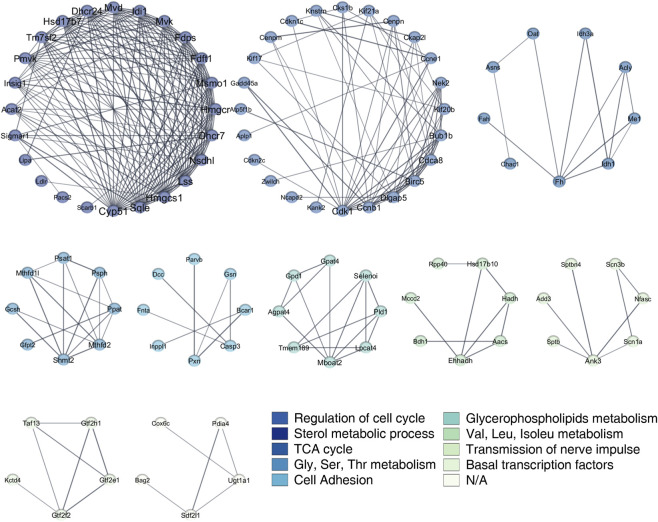
Pro-inflammatory cytokine treatment induces suppression of metabolic and homeostatic interaction modules in oligodendrocytes. Sub-networks identified from a STRING-derived protein–protein interaction (PPI) network. The global network was clustered using the Markov Cluster Algorithm (MCL) to detect groups of highly interconnected nodes. Each sub-network represents a distinct MCL cluster, with nodes corresponding to proteins and edges indicating known or predicted interactions derived from STRING. Within each sub-network, the node positioned at the bottom center denotes the protein with the highest degree. Label size is proportional to node degree, reflecting the relative connectivity of each protein within its cluster. Functional enrichment analysis was performed independently for each sub-network using Gene Ontology Biological Process (GO:BP) and KEGG databases; for each cluster, the most significant and representative enriched term is reported in the adjacent legend.

To place these transcriptional changes into interaction-level context, we performed STRING-derived protein–protein interaction (PPI) network analysis followed by Markov Cluster Algorithm (MCL) clustering. For downregulated genes, PPI clustering revealed discrete suppressed modules centered on sterol metabolic process (a dense cholesterol/sterol biosynthesis cluster), as well as additional clusters annotated as regulation of cell cycle, TCA cycle, glycerophospholipid metabolism, amino-acid metabolism (Gly/Ser/Thr and Val/Leu/Isoleu metabolism), transmission of nerve impulse, basal transcription factors, and a smaller cell-adhesion module (Figure 3). These modules collectively support a coordinated shutdown of anabolic lipid programs together with broader metabolic and homeostatic functions.

**FIGURE 4:**
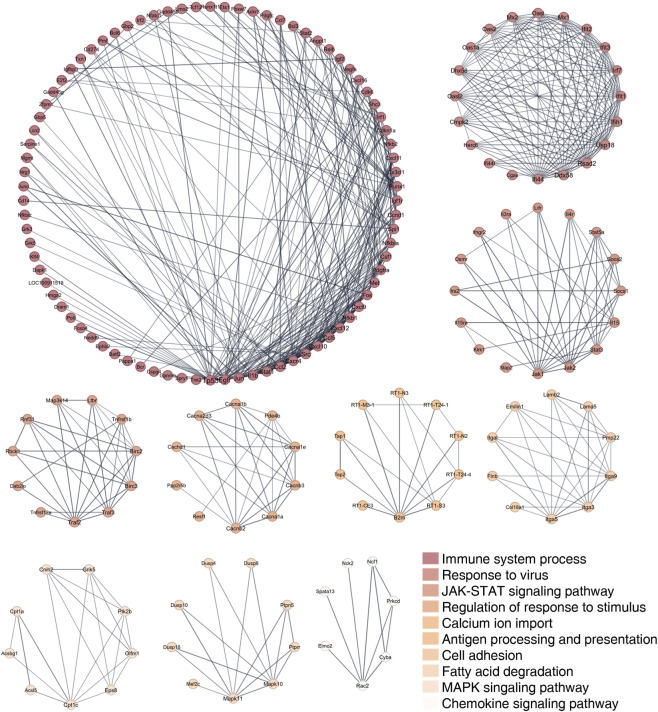
Pro-inflammatory cytokine treatment induces immune-response and cytokine-associated interaction modules in oligodendrocytes. Sub-networks identified from a STRING-derived protein–protein interaction (PPI) network. The global network was clustered using the Markov Cluster Algorithm (MCL) to detect groups of highly interconnected nodes. Each sub-network represents a distinct MCL cluster, with nodes corresponding to proteins and edges indicating known or predicted interactions derived from STRING. Within each sub-network, the node positioned at the bottom center denotes the protein with the highest degree. Label size is proportional to node degree, reflecting the relative connectivity of each protein within its cluster. Functional enrichment analysis was performed independently for each sub-network using Gene Ontology Biological Process (GO:BP) and KEGG databases; for each cluster, the most significant and representative enriched term is reported in the adjacent legend.

In contrast, the upregulated PPI network was dominated by a large “immune system process” module, accompanied by multiple cytokine- and stress-associated clusters including response to virus, JAK–STAT signalling, chemokine signalling, MAPK signalling, regulation of response to stimulus, calcium ion import, antigen processing and presentation, cell adhesion, and fatty acid degradation (Figure 4). Notably, the antigen processing/presentation cluster comprised RT1 family members (MHC class I–related genes), indicating that CTK-2 does not only trigger generic inflammatory signalling in oligodendrocytes but also induces components of immune surveillance pathways.

**FIGURE 5:**
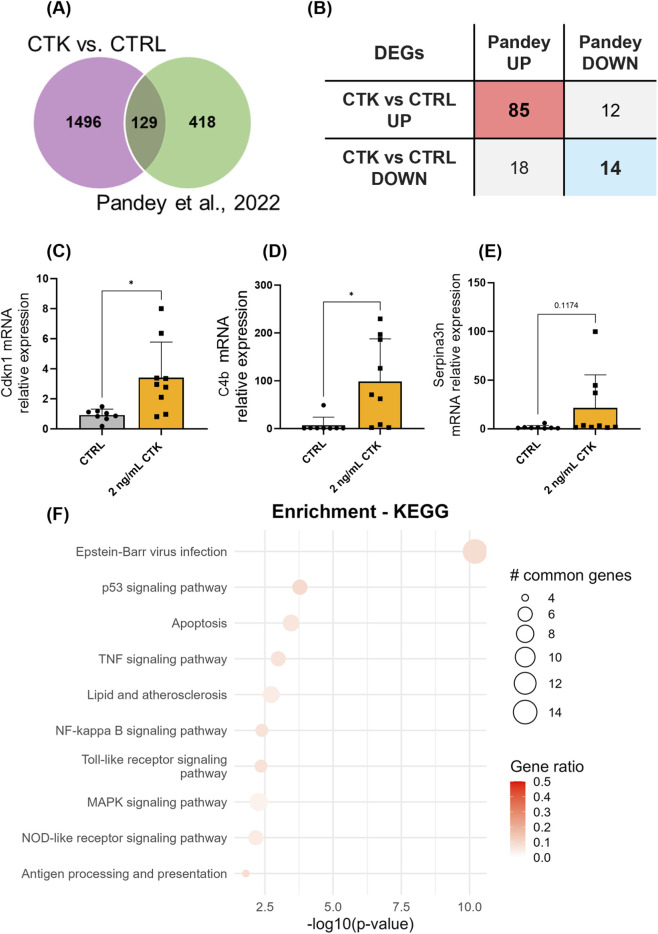
Cytokine cocktail induces inflammatory transcriptional changes common to neurodegenerative disease models and the human brain. **(A)** Venn diagram showing the overlap between differentially expressed genes (DEGs) identified in the CTK-2 versus CTRL after the identification of human orthologs and genes reported in the Pandey et al. (2022) dataset. Numbers indicate unique and shared genes between datasets. **(B)** Contingency table summarizing the directional concordance of the shared gene signature. **(C–E)** Relative mRNA expression (2^-ddCt^) of senescence and disease-associated genes. Graphs show mean ± SD. Dots represent the average of each experiment normalized to the average of the control (CTRL = 1). Unpaired two-tailed t-tests. ‘*’ indicates p value classification when compared to untreated group. **p < 0.01. **(F)** KEGG pathway enrichment analysis of shared upregulated genes, shown as a dot plot. Dot size represents the number of genes associated with each pathway, color indicates gene ratio, and the x-axis reports enrichment significance as −log_10_(P value).

Then, to gain insights on the significance of our CTK model, we compared our CTK-regulated genes with a published cross-species oligodendrocyte disease signature identified in both mouse models and human brains from Alzheimer’s disease and Multiple Sclerosis (Pandey et al., 2022). After converting our DEGs to human orthologs, we identified 129 overlapping genes between the two datasets (Figure 5A; Supplementary Figure S1). Of note, the direction of change was largely concordant: most shared genes were upregulated in both datasets (85 CTK-UP/Pandey-UP) and a substantial fraction were downregulated in both (14 CTK-DOWN/Pandey-DOWN), whereas fewer genes showed opposite regulation (12 CTK-UP/Pandey-DOWN and 18 CTK-DOWN/Pandey-UP) (Figure 5B). These results indicate that our CTK model recapitulates a sizeable transcriptome component of the conserved disease-associated OL transcriptional program.

We next validated three genes previously highlighted as markers of diseased/senescent OLs (Pandey et al., 2022). qPCR confirmed a significant induction of Cdkn1a and C4b in CTK-treated cells, while Serpina3 showed an increasing trend that did not reach statistical significance (Figures 5C–E). Finally, KEGG enrichment analysis restricted to the shared upregulated genes revealed pathways consistent with inflammatory and stress-associated signalling, including TNF/NF-κB, Toll-like receptor, MAPK, JAK–STAT, apoptosis, and p53 signalling, among others (Figure 5F).

Taken together, these data indicate that our model is characterized by a transcriptional response that overlaps in both content and directionality with a conserved neurodegenerative disease–associated OL signature and is accompanied by induction of disease/senescence-linked markers.

### 3.3 The GPR17 agonist galinex rescues OL maturation from CTK-induced differentiation impairment

**FIGURE 6:**
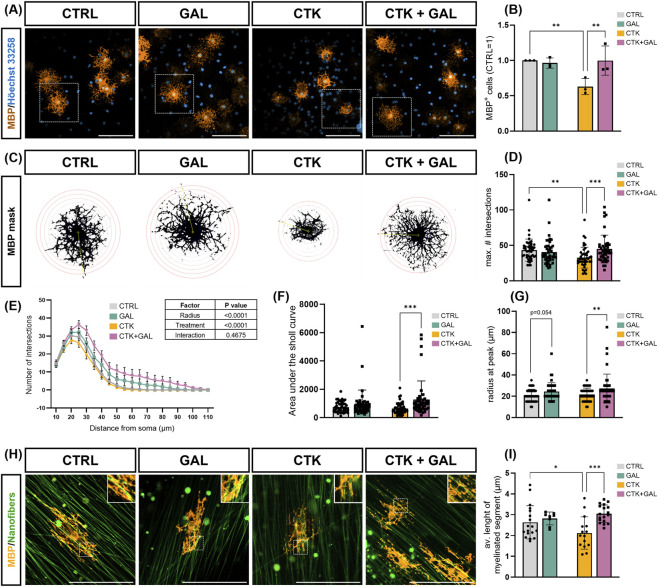
Galinex restores oligodendrocyte maturation, cellular complexity and myelinating capacity following inflammation-mediated dysfunction. **(A)** Representative images (scale bar: 100 µm) of **(B)** the relative proportion of MBP-positive cells after 48 h of treatment with CTK-2, GAL, CTK2+GAL normalized to the average of the control (CTRL = 1). **(C)** Representative images of **(D–G)** Semi-automatized quantification of the maximum number of branch interactions with a concentric ring in the Sholl analysis from MBP-positive cells mask; **(D)** Maximum observed number of intersections with concentric rings per each cell; **(E)** the average number of branch interactions in each concentric ring in the Sholl analysis; **(F)** Area under the curve of **(E)**. Quantification of the diameter of maximum size ring in the Sholl analysis. **(H)** Representative images (scale bar: 100 µm) of **(I)** the average length of myelinated segments per cell of OLs treated on synthetic nanofibers. All graphs show mean ± SD of three independent experiments (n = 3). Dots represent **(A)** the average of each experiment **(D,F,G,I)** single cells. To all, two-way ANOVA with Uncorrected Fisher’s LSD analysis. ‘*’ indicates p value classification with comparisons indicated by lines above columns. *p < 0.05; **p < 0.01; ***; p < 0.001.

To investigate whether galinex (GAL), a GPR17 agonist, could counteract inflammation-induced OL differentiation impairment, we pre-treated OPCs with CTK-2 for 6 h followed by the addition of 10 nM GAL in the medium. As expected, CTK-2 treatment reduced the proportion of MBP-positive cells after 48 h. In contrast, GAL increased the proportion of mature MBP-positive cells to levels comparable to those of untreated control cells (Figures 6A,B), indicating its ability to rescue OL maturation.

To evaluate the impact of inflammatory conditions and GPR17 modulation on OL morphology, we performed Sholl analysis on MBP-expressing cells (Figures 6C–I). Across conditions, Sholl profiles displayed a proximal peak followed by a progressive decline at increasing distances from the Soma. Relative to control cells, exposure to CTK-2 showed a trend toward reduced complexity in the peak/early-decay region but did not reach significance in this dataset when tested per radius (Figures 6E,F). Treatment with GAL yielded a Sholl profile comparable to, and modestly above, control over the peak region and intermediate distances, suggesting enhanced process elaboration within MBP-positive cells. Notably, combined CTK2+GAL treatment increased intersection counts relative to CTK-2 and to control cells across 25–55 µm (FDR-adjusted p < 0.05 across radii) (Figure 6F), consistent with enhanced process elaboration and/or stabilization in MBP-positive OLs under inflammatory challenge. Analysis of single-cell Sholl-derived metrics further indicated increased morphological complexity in CTK2+GAL treated cells, as reflected by higher AUC (Figure 6F) and peak intersection values (Figure 6G) compared with CTK-2, together with a redistribution of branching toward more distal regions.

To further assess OL myelination capacity and obtain insights on the sustained effect of GAL, we cultured differentiating OLs on synthetic nanofibers that mimic axonal substrates. Under these conditions, cells were exposed to CTK-2, GAL alone, or the CTK2+GAL combination and myelination was assessed after 7 days. CTK-2 treatment induced a trend towards reduction in the length of myelinated segments when compared with controls (p = 0.1182), indicating a trend toward impaired OL enwrapping. In contrast, GAL treatment significantly increased the length of myelinated segments compared to CTK-treated cells (Figures 6H,I), indicating that GAL preserves its pro-maturation effect over time and improves the ability of OLs to form myelin-like sheaths under inflammatory conditions.

### 3.4 Galinex elicits early pathway-level remodelling under inflammatory conditions

Although standard DEG filtering for CTK2+GAL vs. CTK-2 did not identify individual genes passing multiple-testing correction, we next performed a pathway-level exploratory analysis using GSEA (Supplementary Table S3), ranking the full expression matrix by the t-statistic for the CTK2+GAL vs. CTK-2 comparison. This approach revealed several significantly enriched KEGG pathways, indicating coordinated but modest transcriptional shifts that may not be captured by single-gene thresholds (Supplementary Figure S2A).

Notably, multiple pathways showed strong negative enrichment, meaning that their member genes are preferentially associated with negative t-statistics and are therefore downregulated in CTK2+GAL compared with CTK-2 (Supplementary Figure S2B). The most significant term was Ribosome (NES = −3.18, padj = 3.12 × 10^−21^), accompanied by mitochondrial/neurodegeneration-related gene sets including Parkinson’s disease (NES = −2.76, padj = 6.42 × 10^−13^) and Oxidative phosphorylation (NES = −2.62, padj = 4.59 × 10^−10^), as well as Huntington’s disease and Alzheimer’s disease. In parallel, proteostasis-associated pathways such as Proteasome, Protein processing in endoplasmic reticulum, and Protein export were also negatively enriched, suggesting an early GAL-associated downshift of translation/mitochondrial and protein-handling programs in cytokine-treated oligodendrocytes. In contrast, Lysine degradation was the only significantly positively enriched pathway (NES = +1.91, padj = 0.0189), consistent with a selective relative increase of amino-acid catabolic gene expression in CTK2+GAL versus CTK-2 (Supplementary Figure S2B). Overall, these results support the idea that, at this early time point, GAL does not induce large changes at the single-gene level but is associated with coherent pathway-level remodelling in the context of inflammatory stimulation (Supplementary Figure S2B).

GAL-alone treatment was also evaluated compared to untreated cells. GSEA analysis showed that GAL alone was transcriptionally active, modulating pathways partially overlapping with those observed in the CTK2+GAL vs. CTK-2 comparison, including ribosome, proteasome, oxidative phosphorylation, and protein processing in the endoplasmic reticulum. GAL alone also positively enriched phosphatidylinositol and phospholipase D signalling pathways, consistent with engagement of receptor-associated signalling (Supplementary Figure S3). These data support the view that GAL is biologically active under control conditions, although its functional pro-maturation effect did not emerge in a more physiological context.

## 4 Discussion

Chronic neuroinflammation represents a major barrier to effective remyelination in several neurodegenerative disorders, shaping the balance between tissue repair and progressive degeneration (Klotz et al., 2023; Abdelhak et al., 2025). While immunomodulatory therapies have substantially improved disease control, they fail to directly restore myelin integrity, particularly in contexts of persistent, low-grade inflammation (Klotz et al., 2023) and despite somewhat changing disease progression, they do not promote functional recovery. Increasing evidence indicates that in neurological disorders, where inflammation strongly contributes to neurodegeneration, OL lineage cells are not merely passive targets of inflammatory damage but actively respond to inflammatory cues by adopting altered transcriptional and functional states that can compromise their differentiation and myelinating capacity (Boccazzi et al., 2022; Festa et al., 2025). In this context, defining how non-cytotoxic inflammatory stimuli alter oligodendrocyte differentiation programs is essential to understand remyelination failure and to identify strategies to restore myelin-forming competences.

Despite previous studies have investigated the effects of pro-inflammatory cytokines on maturation and myelination of isolated OLs or OPCs, most of them have relied on single cytokines often used at supraphysiological concentrations that can be overtly toxic, and may not capture the sublethal inflammatory environment in which OPCs are still present but fail to differentiate and remyelinate (Bonora et al., 2014; De Nuccio et al., 2015; Desu et al., 2021). To overcome these limitations, we established and characterized an *in vitro* model of inflammation-induced oligodendrocyte differentiation impairment by using pro-inflammatory cytokines (TNF-α, IL-1β, and IFN-γ) commonly upregulated in neuroinflammatory disease (Rossi et al., 2014; Kallaur et al., 2017; Shen et al., 2019). Our results demonstrate that exposure to a pro-inflammatory cytokine cocktail, at an optimized concentration of 2 ng/mL, elicits a robust inflammatory response, evidenced by the upregulation of chemokines Ccl2, Ccl5, and Cxcl10, without compromising cell viability, thereby avoiding the cytotoxicity observed at higher dosages (6 and 20 ng/mL). The robust upregulation of these mediators is consistent with previous findings obtained with higher concentrations of cytokines (Desu et al., 2021), suggesting that OPCs are highly sensitive to inflammation. Crucially, this specific inflammatory milieu was associated to a distinct maturation arrest: while we observed a significant reduction in mature MBP-positive oligodendrocytes, the population of BCAS1-positive pre-myelinating cells remained unaffected. This phenotype, further corroborated by the sustained expression of the immature markers Gpr17 and Cspg4, suggests that the inflammatory stimulus halts the differentiation program specifically at the transition from the pre-myelinating to the myelinating stage, rather than by depleting the early progenitor pool.

At transcriptomic level, our analysis revealed that CTK-2 induced a transcriptional program in OLs that shows substantial convergence with the disease-associated oligodendrocyte (DAO) states previously identified across human and mouse models (Pandey et al., 2022) (Figure 5). In particular, as expected, CTK treatment recapitulates key features of the pro-inflammatory “DA1” program, including robust induction of cytokine-mediated signalling, chemokines, Toll-like receptors, and antigen-presentation-related transcripts (Figures 5F, 2F–H), indicating a shift toward an immunogenic phenotype. In parallel, the upregulation of stress-response genes such as Tp53 and Rras aligns with characteristics of the “DA2” state, indicating that CTK-2 exposure engages both inflammatory and stress-associated transcriptional modules. Consistent with this overlap, several hallmark DA1/DA2 genes (including C4b, Serpina3n, and Cdkn1a) were also induced by CTK-2 (Figures 5C–E). PPI network analysis further confirms that CTK-induced immune-related genes are organized into a coherent and highly interconnected module encompassing cytokine signalling, stress-associated pathways, and antigen processing and presentation (Figures 3, 4). The inclusion of RT1 family members within this network supports the engagement of immune surveillance–associated machinery in CTK-treated oligodendrocytes. Collectively, these findings support the hypothesis that CTK-2 treatment recapitulates transcriptional states that parallel maladaptive responses observed across neurodegenerative conditions and strongly validates our *in vitro* model for assaying both inflammatory stimuli and the ability of pharmacological agents to contrast their deleterious effects on neurodegeneration.

Beyond immune reprogramming, our transcriptomic data indicate that our model is characterized by a coordinated suppression of lipid anabolic and bioenergetic programs that are essential for oligodendrocyte maturation and myelin maintenance (Figure 3). Oligodendrocytes are uniquely dependent on lipid synthesis, particularly cholesterol, fatty acids, and sphingolipids, for membrane elaboration and myelin sheath assembly, and disruptions in these pathways have been implicated in demyelinating disease and impaired remyelination capacity (Marangon et al., 2022; Marangon et al., 2020). Accordingly, the broad downregulation of genes encoding rate-limiting enzymes of fatty acid and cholesterol synthesis in our CTK model mirrors the inflammation-induced lipid metabolic dysfunction found in all DAO clusters (Pandey et al., 2022). At the network level, the suppression of TCA cycle and amino-acid metabolic modules suggests that CTK-2 triggers a broader metabolic reprogramming beyond canonical lipid biosynthesis. This finding is consistent with the notion that, during differentiation, oligodendrocyte metabolic states are finely reprogrammed to support biosynthesis, redox balance, and interactions with axonal metabolism, such that perturbations in these pathways may compromise not only myelin production but also cellular resilience under stress (Han and Cheng, 2025; Narine et al., 2022). These data are consistent with previous results demonstrating that, after reaching the O4 differentiation stage, rewiring of OL metabolism to address terminal maturation is driven by the downregulation of the GPR17 receptor, whose inappropriate prolonged overexpression under inflammatory conditions is indeed responsible for OL maturation block and impaired myelination (see also below). Altered inflammation-driven metabolic reprogramming of oligodendrocytes provides a mechanistic framework to interpret remyelination failure in smouldering neuroinflammatory contexts.

Up to date, several drugs have been used to stabilize and prevent worsening of neurodegenerative diseases, including monoclonal antibodies and immunomodulatory drugs. In this clinical context, these so-called disease-modifying drugs are able to reduce the frequency and severity of relapses and slow down the progression of the disease (Dobson and Giovannoni, 2019; Rafii and Aisen, 2025). However, no current therapies were shown to restore remyelination and rescue from neurodegeneration, partially due to the fact that none specifically targets OLs (Klotz et al., 2023). This represents a critical therapeutic gap, since, in the adult CNS, oligodendrocytes do retain the intrinsic potential to mature and remyelinate, a capacity that is strongly hampered by the non-permissive inflammatory environment (Coppolino et al., 2018; Starost et al., 2020; Mozafari et al., 2020). In this context, we focused on GPR17, a receptor essential for OL maturation that is known to become dysregulated under pathological conditions (Lecca et al., 2020). In our model, we observed an upregulation of GPR17 gene expression following inflammatory stimulation (Figure 1K). This aligns with previous evidence of aberrant GPR17 accumulation across various CNS disorders characterized by inflammation such as traumatic brain injury (Boda et al., 2011), stroke (Gelosa et al., 2019), neurodegeneration (Bonfanti et al., 2020), or demyelinating models (Coppolino et al., 2018; Ch et al., 2009; Seyedsadr and Ineichen, 2017). This dysregulation is also observed in MS and stroke human brains, where GPR17-positive cells accumulate at the borders of lesions and, most importantly, in the inflamed still normal-appearing tissue, suggesting that prompt GPR17-based interventions may help maintaining myelin integrity (Angelini et al., 2021; Raffaele et al., 2025). Therefore, targeting GPR17 signalling represents a strategy to overcome inflammation-induced maturation arrest and promote remyelination.

To pursue this therapeutic avenue, we investigated the effects of galinex (GAL), a selective GPR17 agonist (Parravicini et al., 2020). While previous work demonstrated its efficacy in delaying disease symptoms in the *in vivo* experimental autoimmune encephalomyelitis (EAE) mouse model, the specific cellular mechanisms exerted by GAL on OLs under inflammatory conditions remained to be further elucidated in a more controlled environment.

We first observed that GAL induced a morphological recovery of inflammation-exposed differentiating OLs. Transition from a precursor state to a myelinating phenotype requires profound cytoskeletal rearrangement, since OLs must extend and ramify their processes to become competent for myelination (Osanai et al., 2022; Shimizu et al., 2018). In our culture system, the pro-inflammatory CTK-2 cocktail significantly impaired this arborization, as shown by a reduction in the number of cell branches (Figure 2I), indicating that inflammation locks OLs into a simplified, retracted state. Notably, GAL treatment not only increased the number of MBP-positive cells (Figures 6A,B), as expected for a promyelinating agent, but robustly rescued their morphological complexity, promoting the “arborized phenotype” necessary for functional maturation. To analyse in detail if the observed morphological rescue translated into functional wrapping capacity, we utilized a nanofiber assay. By plating OLs onto synthetic nanofibers, we provided a physical substrate mimicking axons (Baydyuk et al., 2020; Duncan et al., 2021) while maintaining a purely OL-intrinsic environment. We observed that GAL treatment restores the length of myelinated segments on the nanofibers (Figures 6H,I). Altogether, these results indicate that the GPR17 agonist does not only restore the structural complexity of OLs, but also supports their functional ability to synthesize and wrap myelin around a target, even in the presence of inflammatory cytokines, suggesting that its pro-differentiative effect can persist long enough to influence later stages of OL maturation.

As mentioned, the observation that a GPR17 agonist restores proper OL maturation may appear counterintuitive, as physiological GPR17 expression typically restrains their differentiation to allow cells to prepare to functional maturation and coordinate myelination timing. We have previously demonstrated that endogenous GPR17 agonists can promote maturation in specific *in vitro* contexts (Fumagalli et al., 2011; Coppi et al., 2013). More importantly, we have shown that sustained activation of GPR17 by agonists leads to rapid receptor desensitization and subsequent internalization (Fratangeli et al., 2013; Daniele et al., 2014; Fumagalli et al., 2015; Daniele et al., 2011). Consequently, we propose that in our experimental setting, GAL may act as a “functional” antagonist. By inducing receptor internalization and downregulation directly at the oligodendrocyte membrane, GAL could mitigate the differentiation arrest, allowing cells to progress toward a mature, myelinating phenotype despite the presence of inflammatory cytokines.

Building on these functional observations, we asked whether GAL co-treatment also reshapes the transcriptional programs suppressed by CTK. Because GAL-associated effects were modest at individual-gene level at the observed time of 24 h, we adopted a pathway-level strategy and performed GSEA on ranked gene lists, which is able to detect coordinated shifts distributed across several genes. The GSEA profile of the CTK2+GAL vs. CTK-2 comparison showed negative enrichment across several KEGG pathways (Supplementary Figure S2). In particular, pathways related to ribosomal/translation machinery and mitochondrial respiration emerged as the most affected. Alongside these mitochondrial and translational programs, CTK2+GAL co-treatment was also associated with reduced enrichment of proteostasis-related pathways, including proteasome function and ER-associated protein processing/export, pointing to an early adjustment of protein synthesis and handling under inflammatory stress. Mechanistically, these pathway-level signatures fit with the broader concept that modulation of GPR17 signalling affects the metabolic state of oligodendrocytes during differentiation, with changes that likely precede the morphological and functional features associated with OL maturation. We have previously demonstrated that, in differentiating OLs, GPR17 silencing by a biotechnological approach promoted OL differentiation alongside a progressive metabolic rewiring characterized by an increase in Krebs cycle metabolites and reduction in glucose and lactate, followed by shifts in several lipid classes (free fatty acids, ceramides, acyl-alkyl-phosphatidylcholine, and phosphatidylinositol) (Marangon et al., 2022). Transcriptomics analysis in the same study also indicated that GPR17 silencing increased the expression of LXRα nuclear lipid receptor and its target genes, which suggests that suppressing GPR17 signalling reorganizes energy utilization and lipid handling in a manner permissive for maturation, reflecting distinct temporal windows and manners of GPR17 modulation, rather than opposing biological outcomes (Marangon et al., 2022).

To our knowledge, this study provides the first evidence that agonist-based GPR17 modulation can foster OPC differentiation under inflammatory conditions; however, several limitations should be acknowledged. Our study is based on an *in vitro* approach designed to characterize oligodendroglial responses to defined subtoxic inflammatory stress. This reductionist system does not recapitulate the full cellular and temporal complexity of the *in vivo* neuroinflammatory environment, where microglia, astrocytes, peripheral immune cells and chronic lesion dynamics actively shape demyelination and repair. However, it allowed us to directly assess the effect of cytokine exposure on oligodendrocyte maturation and to test whether GPR17 pharmacological modulation acts on oligodendroglial cells under inflammatory stress. Furthermore, our experiments focus on early time points (24–48 h), so the transcriptional and morphological changes we report reflect acute responses and may not predict longer-term outcomes. Finally, although previous works support agonist-induced GPR17 desensitization/internalization, receptor trafficking was not directly tested here. Future work should test longer treatment windows to resolve delayed transcriptional programs, include orthogonal assays of receptor dynamics, and evaluate GPR17 modulation in more complex inflammatory models.

Globally, our results suggest that the pro-maturation effect of GAL is unlikely to depend on a broad suppression of the inflammatory transcriptional response. Rather, GAL appears to promote OL maturation-associated programs despite the persistence of an inflammatory state. This point is particularly relevant because pharmacological modulators specifically targeting oligodendrocytes are still missing. *In vivo*, such an approach would likely complement, rather than replace, immunomodulatory or disease-modifying therapies aimed at controlling neuroinflammation.

## Data Availability

The data presented in the study are deposited in the GEO profile repository, accession number GSE331058.
