# Supplementary material for: The GPR17 agonist galinex restores oligodendrocyte maturation under inflammatory conditions
Source: Front Pharmacol. 2026 Jun 5;17:1838997. doi: 10.3389/fphar.2026.1838997 (PMC13279327; doi:10.3389/fphar.2026.1838997)
Supplement: Supplementary file 3 [file Table1.docx]

***Supplementary Material***

**Supplementary table 1. List of primer pairs used for qRT-PCR.**

| Gene | Forward primer | Reverse primer |
| --- | --- | --- |
| Ccl-2 | TGCTGCTACTCATTCACTGGC | TGATCCCAATGAGTCGGCTG |
| Ccl-5 | CTCATTCCTGGGAGGGCATA | TGTACAGAGCCTGTGAAGAGC |
| Cxcl-10 | TGCAAGTCTATCCTGTCCGC | CTCTGCTGTCCATCGGTCTC |
| Cdkn1 | TGTCCGACCTGTTCCACACA | CGTCTCAGTGGCGAAGTCAA |
| Serpina3n | ACAACACACCAGGGAAGTGG | CACAAGGCGGCGGGTCATCTTCT |
| C4b | GCCCTGGGTAAAGTGAATAC | TCTGCTTCCTCCATCTCTC |
| Cnp | CCAACAGGATGTGGTGAGGA | GATGAGGGCTTGTCCAGGTC |
| Cspg4 | TGTTCTCACACAGAGGAGCC | GGCCACCACTCGGAAGAAAT |
| Gpr17 | ACAATGCCTCCCTGGCTTAC | CAGAGCGTTGCCCACAAAAG |
| Mbp | CTTCCTCCCAAGGCACAGAG | GTGTGTGAGTCCTTGCCAGA |
| Rpl13a | TCCGAAGAAGGGAGACAGTT | CTTCTCCTCTTCCGTGGATGG |
