# Supplementary figures and images for: The GPR17 agonist galinex restores oligodendrocyte maturation under inflammatory conditions
Source: Front Pharmacol. 2026 Jun 5;17:1838997. doi: 10.3389/fphar.2026.1838997 (PMC13279327; doi:10.3389/fphar.2026.1838997)

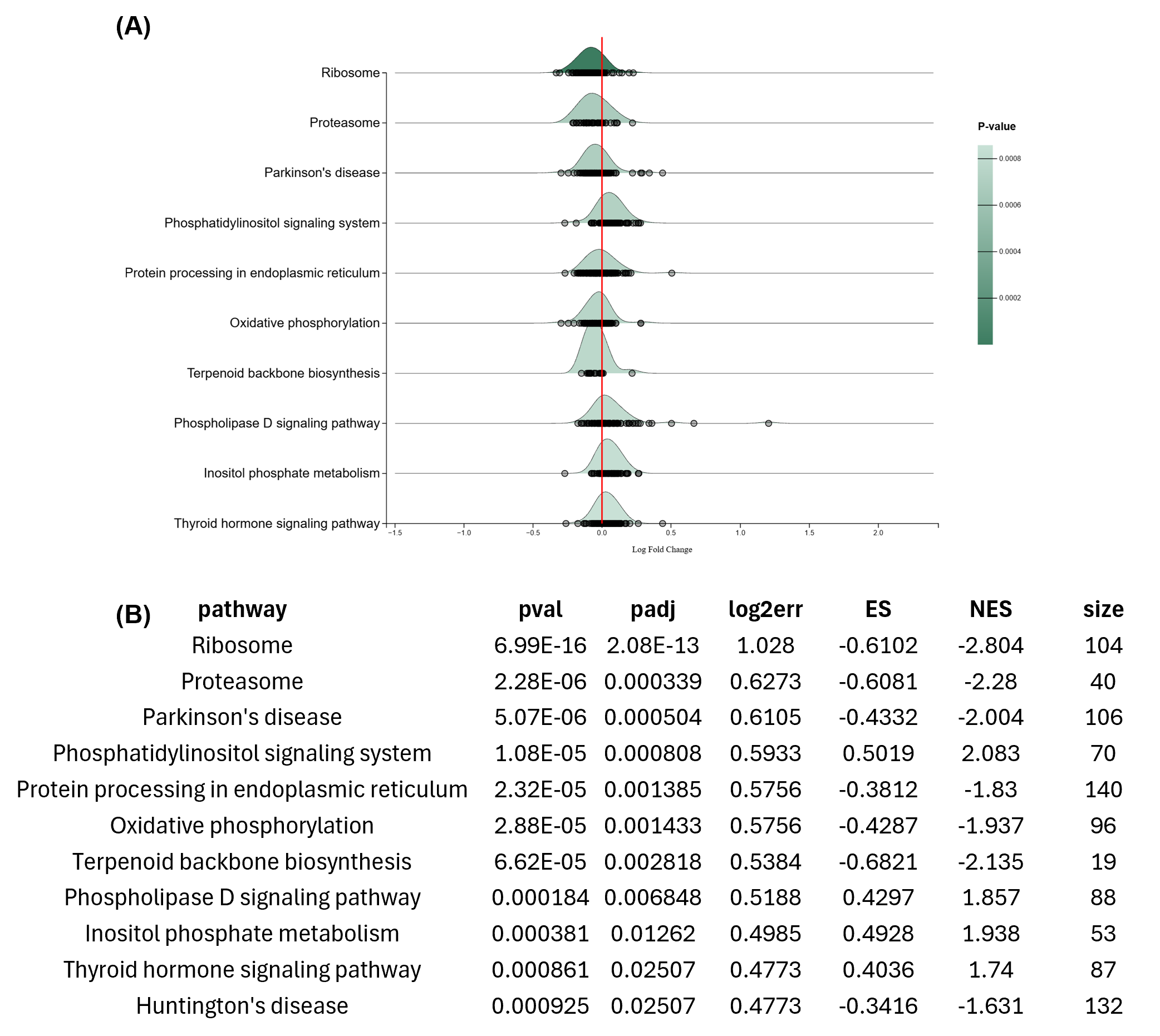

Supplement: Supplementary file 4 [file Image3.tif]

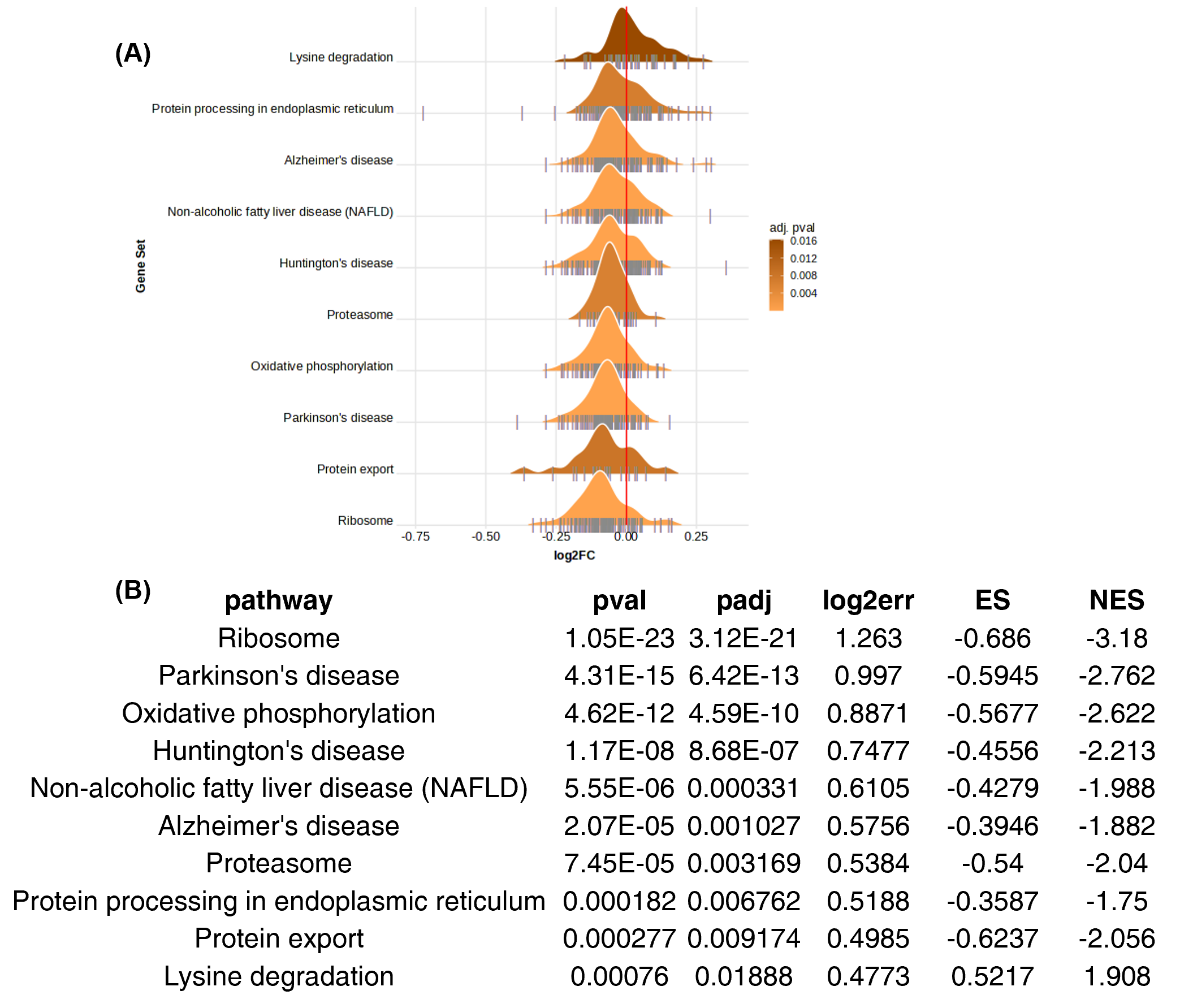

Supplement: Supplementary file 5 [file Image2.tif]

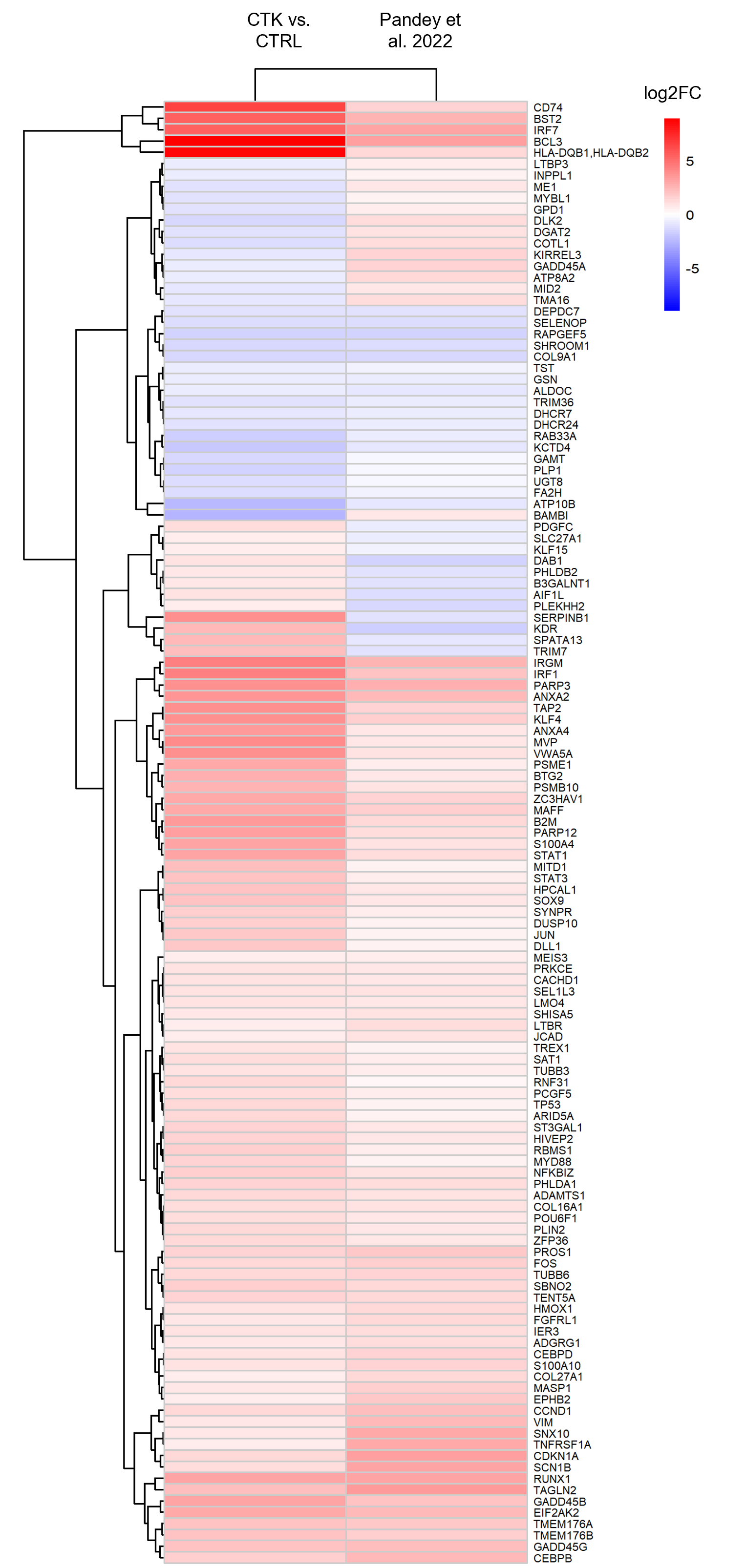

Supplement: Supplementary file 6 [file Image1.tif]
